# Unravelling Magnetic Nanochain Formation in Dispersion for In Vivo Applications

**DOI:** 10.1002/adma.202008683

**Published:** 2021-05-07

**Authors:** Nileena Nandakumaran, Lester Barnsley, Artem Feoktystov, Sergei A. Ivanov, Dale L. Huber, Lisa S. Fruhner, Vanessa Leffler, Sascha Ehlert, Emmanuel Kentzinger, Asma Qdemat, Tanvi Bhatnagar‐Schöffmann, Ulrich Rücker, Michael T. Wharmby, Antonio Cervellino, Rafal E. Dunin‐Borkowski, Thomas Brückel, Mikhail Feygenson

**Affiliations:** ^1^ Forschungszentrum Jülich GmbH Jülich Centre for Neutron Science JCNS and Peter Grünberg Institut PGI, JARA‐FIT 52425 Jülich Germany; ^2^ Lehrstuhl für Experimentalphysik IVc RWTH Aachen University 52056 Aachen Germany; ^3^ Australian Synchrotron ANSTO Clayton 3168 Australia; ^4^ Forschungszentrum Jülich GmbH Jülich Centre for Neutron Science (JCNS) at Heinz Maier‐Leibnitz Zentrum (MLZ) 85748 Garching Germany; ^5^ Materials Physics and Applications Division: Center for Integrated Nanotechnologies Los Alamos National Laboratory Los Alamos NM 87545 USA; ^6^ Center for Integrated Nanotechnologies Sandia National Laboratories Albuquerque NM 87123 USA; ^7^ Institute of Physical Chemistry RWTH Aachen University Landoltweg 2 52056 Aachen Germany; ^8^ Forschungszentrum Jülich GmbH Jülich Centre for Neutron Science (JCNS‐1) and Biological Matter (IBI‐8) 52425 Jülich Germany; ^9^ Forschungszentrum Jülich GmbH Ernst Ruska‐Centre for Microscopy and Spectroscopy with Electrons and Peter Grünberg Institute 52425 Jülich Germany; ^10^ PETRA III Deutsches Elektronen‐Synchrotron DESY 22607 Hamburg Germany; ^11^ Swiss Light Source Paul‐Scherrer‐Institut Villigen PSI 5232 Switzerland

**Keywords:** in vivo applications, magnetic nanoparticles, nanochains, neutron scattering, reverse Monte Carlo simulations

## Abstract

Self‐assembly of iron oxide nanoparticles (IONPs) into 1D chains is appealing, because of their biocompatibility and higher mobility compared to 2D/3D assemblies while traversing the circulatory passages and blood vessels for in vivo biomedical applications. In this work, parameters such as size, concentration, composition, and magnetic field, responsible for chain formation of IONPs in a dispersion as opposed to spatially confining substrates, are examined. In particular, the monodisperse 27 nm IONPs synthesized by an extended LaMer mechanism are shown to form chains at 4 mT, which are lengthened with applied field reaching 270 nm at 2.2 T. The chain lengths are completely reversible in field. Using a combination of scattering methods and reverse Monte Carlo simulations the formation of chains is directly visualized. The visualization of real‐space IONPs assemblies formed in dispersions presents a novel tool for biomedical researchers. This allows for rapid exploration of the behavior of IONPs in solution in a broad parameter space and unambiguous extraction of ​the parameters of the equilibrium structures. Additionally, it can be extended to study novel assemblies formed by more complex geometries of IONPs.

## Graphical abstract

Biocompatible iron oxide nanoparticles in dispersion can be controlled to form flexible and reversible chains in the presence of a magnetic field, which is useful for biomedical applications. The role of control parameters is determined and these novel assemblies formed in dispersions are visualized using advanced scattering methods and reverse Monte Carlo simulations.

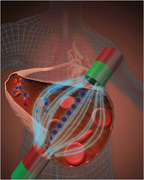

## 1 Introduction

Biocompatible iron oxide nanoparticles (IONPs) are well suited for in vivo applications such astargeted drug delivery, contrast agents for magnetic resonance imaging (MRI), or hyperthermia treatment.^[^ ^1^, ^2^, ^3^, ^4^, ^5^, ^6^, ^7^ ^]^ The IONPs in the size range between 10 and 100 nm are considered optimal for intravenous injection and prolonged blood circulation.^[^ ^8^ ^]^ Control and manipulation of IONPs with applied magnetic fields are essential for those applications. IONPs can assemble into extended structures when magnetic field is applied. Assemblies of IONPs are more appealing for in vivo applications than single particles because of their larger magnetic moment, which are better controlled by smaller magnetic fields. For example, IONPs conjugated with virus assemble are used as magnetic viral nanosensors for selective detection of viruses.^[^ ^9^ ^]^ In the presence of a magnetic field spherical IONPs are shown to self‐assemble into a 1D, 2D, or 3D structures.^[^ ^10^, ^11^, ^12^, ^13^ ^]^ However, 1D chains offer a number of advantages for in vivo applications. They have a better mobility while traversing through circulatory passages, as well as a higher targeting accuracy for drug delivery.^[^ ^14^ ^]^ Flexible 1D chains with an active propulsion system are shown to have an enhanced contrast for MRI applications, which is easier to interpret as compared to 2D/3D assemblies.^[^ ^14^, ^15^ ^]^ 1D chains have become more and more critical not only for medical applications, but for fabricating of nanocircuits, waveguides, and logic computations devices.^[^ ^15^, ^16^, ^17^ ^]^ Yet, many studies are devoted to complex 2D/3D structures, while experimental studies of “simpler” 1D structures are somewhat limited.^[^ ^18^, ^19^, ^20^ ^]^

The self‐assembly of IONPs is driven by a delicate balance between attractive (e.g., magnetic dipolar, van der Waals) and repulsive (e.g., electrostatic, steric) interactions.^[^ ^21^ ^]^ The strength of these interactions depends on several parameters such as size, particle geometry, concentration, composition, ligand shell thickness, temperature, and magnitude of the applied magnetic field. The theoretical foundation for 1D chains was developed as early as the 1970s in the pioneering work by de Gennes and Pincus.^[^ ^22^ ^]^ They predicted formation of chains in zero magnetic field in solution. But it was not until late 1990s, when the first numerical studies of chain formation using Monte Carlo simulations revealed that the chains formed by nanoparticles are flexible and may bend, break, and recombine making them similar to “living polymers.”^[^ ^23^ ^]^ Nearly 40 years later successful direct imaging of chains in zero field in dried magnetite colloid was possible by using transmission electron microscopy at cryogenic temperatures (cryoTEM).^[^ ^11^, ^24^, ^25^ ^]^ Moreover, template‐assisted chains were reported by various groups using conventional microscopy techniques.^[^ ^26^, ^27^ ^]^ Several transmission electron microscopy (TEM) studies have provided experimental evidences of chain formation; however, the spatial confinement of chains on a TEM grid or a substrate does not replicate the actual environment of IONPs for in vivo applications. Chain formation was also studied in solutions by X‐ray and neutron scattering methods. Klokkenburg et al., showed that IONPs formed 3D‐ordered structures in dispersions in an applied field of 1 T via intermediate chain formation.^[^ ^10^ ^]^ In some cases, no chain formation was observed at all and single crystal‐like 3D assembly of IONPs was formed in magnetic field of 2.2 T.^[^ ^12^ ^]^ Barrett et al., demonstrated presence of short chain segments 3–4 particles for cobalt nanoparticles dispersions.^[^ ^28^ ^]^ In this work, we use scattering methods to study chain formation by spherical IONPs in a dispersion at room temperature, simulating an environment which is more relevant for in vivo applications. Investigations were carried out on both commercial and specifically synthesized IONPs with various diameters (5–27 nm) and concentrations (0.66–44 mg mL^−1^) subjected to magnetic fields of 0–2.2 T. We combine scattering data with advanced Reverse Monte Carlo (RMC) simulations to provide real‐space distributions of self‐assembled IONPs at various applied magnetic fields, including zero‐field for IONPs in dispersion. Our method is akin to direct visualization of IONPs deposited on a substrate using electron microscopy.

## 2 Results and Discussion

**Figure 1:**
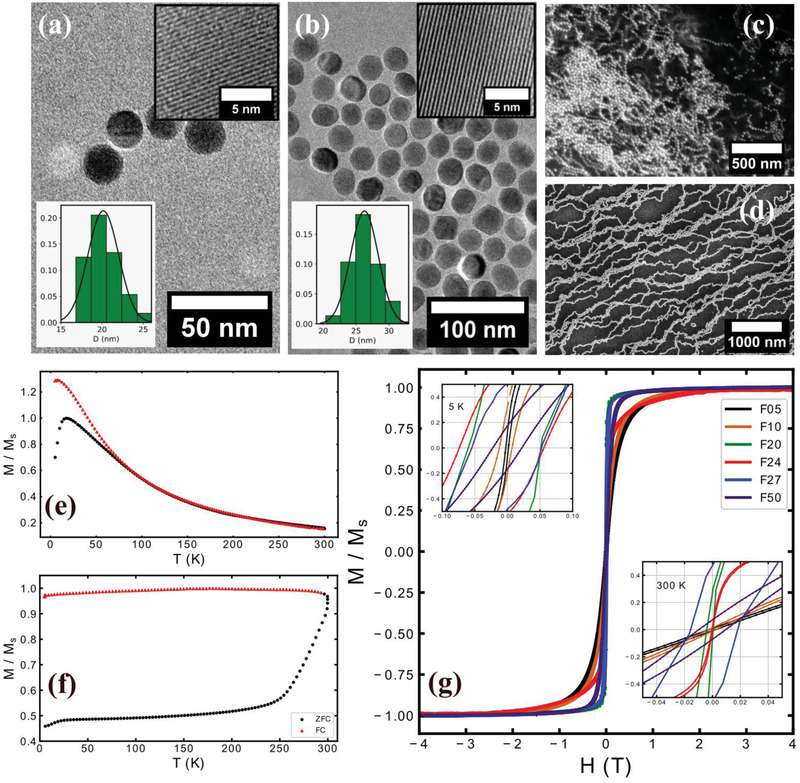
a,b) epresentative TEM images of F20 (a) and F27 (b) nanoparticles. The insets show high resolution images and derived size distributions. c,d) SEM images of F27 nanoparticles drop cast (c) and spin coated (d) on a silicon substrate. e) ZFC/FC curves of F10 measured at 0.01 T. f) ZFC/FC curves of F27 at 0.01 T. g) *M–H* curves of IONPs at 300 K. The insets show the same *M–H* curves at low *H*, at 5 K (top) and at 300 K (bottom).

An overview of the samples used in this study (**Table** **1**) includes commercially synthesized particles from NNlabs (F05, F10), OceanNanotech (F24), Sigma‐Aldrich (F50) and specifically synthesized samples (F20, F27). F20 and F27 samples were synthesized at Center for Integrated Nanotechnologies, Sandia National laboratories and Los Alamos National Laboratory (LANL) using an extended LaMer mechanism.^[^ ^29^ ^]^ This is a method of choice for synthesizing monodisperse nanoparticles of bigger sizes in large quantities suitable for in vivo applications. The crystal planes are clearly visible in the representative high resolution TEM images of F20 and F27 (**Figure** **1a**,b) indicating the high crystalline order of the specifically synthesized IONPs. TEM images of other samples are shown in Figure S1 in the Supporting Information. We carried out synchrotron X‐ray PDF measurements on selected IONPs in order to probe their composition and local crystal structure. The best model for F20 assumes a mixed composition of 23% maghemite and 77% magnetite (mass fractions), while the model for F27 is a mixture of 48% maghemite and 52% magnetite. We observe some evidence of local disorder or antiphase boundaries in the F20 and F27 samples, manifested by considerable deviations of the model from experimental data at *r* < 12 Å (Figure S2, Supporting Information). The size and composition dependent magnetic properties of IONPs are characterized with field‐ and temperature‐dependent DC magnetization measurements and compared with a reference (F50).

**Table 1:** Summary of samples used in this work, where *D* is diameter and Δ*D* is polydispersity; *T*_B_ is blocking temperature. Exchange bias (*H*_EB_) and coercive (*H*_C1_) fields are obtained at 5 K. Standard deviation for both values is 15 Oe. N* is the aggregation parameter calculated for concentration 22 mg mL^−1^ in saturation magnetic field

Sample ID	*D* [nm]	Δ*D* [%]	*T*_B_ [K]	*H*_EB_ [Oe]	*H*_C1_ [Oe]	N*
F05	5.2 ± 0.6	12	10	0	16	0.07
F10	9.5 ± 0.8	9	18	27	70	0.22
F20	20 ± 1.8	9	250	43	513	≈10^3^
F24	24 ± 2	9	300	90	561	≈10^6^
F27	27 ± 2	8	>300	11	523	≈10^10^
F50 (ref)	50 ± 25	50	>300	0	220	≈10^70^

Field‐cooled (FC) and zero‐field‐cooled (ZFC) magnetizations as a function of temperature were used to measure the blocking temperature (*T*_B_) of our particles for different concentrations (Figure 1; Figures S3–S5, Supporting Information). F10 sample (Figure 1e) shows *T*_B_ of about 18 K, while *T*_B_ for F27 sample is above 300 K (Figure 1f). Despite the magnetite phase present in our IONPs as shown with *x*PDF, only F50 sample revealed a kink in *M*(*T*) which we interpret as Verwey transition at around 100 K in ZFC magnetization (Figure S3c, Supporting Information), lower than the nominal temperature of 125 K. A visible Verwey transition is suppressed in IONPs with diameters smaller than 50 nm, in agreement with previous report.^[^ ^30^ ^]^ The measured saturation field was around the bulk value of 0.2 T for all samples, and 0.5 T for F24 at 300 K (Figure 1g). *M–H* curves revealed the finite exchange bias field for all samples at 5 K, when cooled down in 1 T from 300 K (top inset Figure 1g). The experimental *T*_B_ is higher than the theoretical one for noninteracting IONPs of the same size and composition (Figure S6, Supporting Information). The increased *T*_B_ is due to dipole–dipole interactions between IONPs, which become stronger for larger particles.^[^ ^30^ ^]^ These dipole–dipole interactions are dominant in F27 due to a large size and are vital driving forces in formation of chains. Using scanning electron microscopy (SEM), chain formation can be directly observed for F27 IONPs drop cast and spin coated on a substrate at ambient conditions. Drop‐cast samples indicates the presence of chains even in zero field for F27 sample (Figure 1c) in agreement with previous observations of zero‐field chains with cryoTEM.^[^ ^24^ ^]^ The chains are aligned and disentangled by a combination of centrifugal forces and surface tension when the sample is spin coated (Figure 1d).

Simulations employing Langevin approximations have shown that the aggregation parameter $N^{*}\;=\sqrt{\phi_{o}e^{\gamma -1}\;}\;$, which is a combination of dipolar coupling strength γ and the volume fraction *Φ*_o_, is the main parameter controlling chain formation in dilute systems.^[^ ^31^ ^]^ Three cases can be distinguished: (i) N* ≤ 1 results in no chain formation; (ii) a N* > 1 describes an equilibrium state with a finite chain length, or (iii) non‐equilibrium state with a power‐law increase of chain lengths when magnetic field is applied. N*is calculated in Table 1 for the concentration of 22 mg mL^−1^ for all samples. The concentration dependence of N* is depicted in Figure S7 in the Supporting Information.

**Figure 2:**
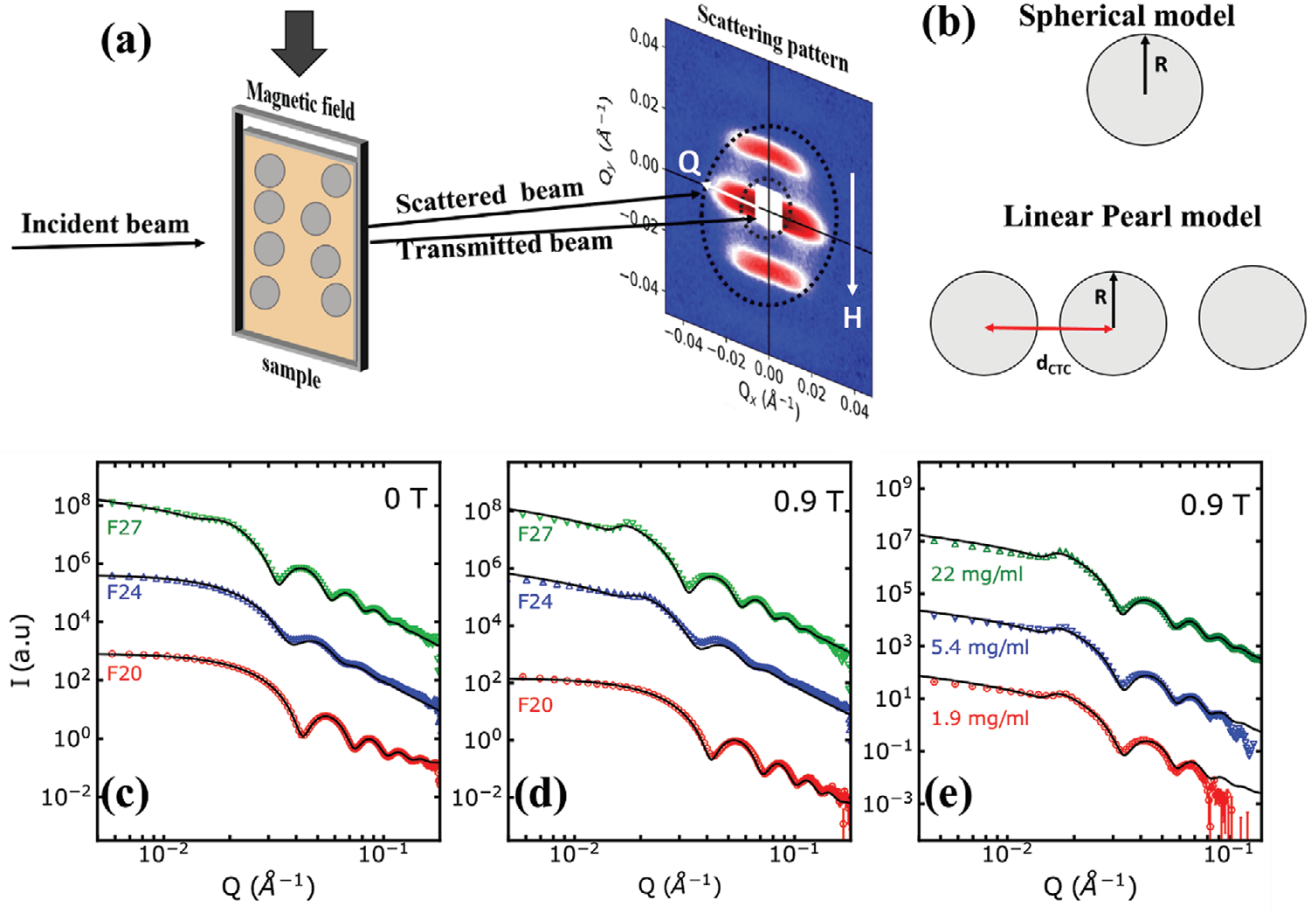
a) Schematic of the SAXS/SANS setup. The dotted lines on the detector indicate the annulus in which the data were radially averaged. b) Schematic of the spherical form factor and linear pearl model. c,d) Radially averaged SAXS data collected at 0 T (c) and 0.9 T (d) for F20, F24, and F27 samples. e) SAXS data collected at 0.9 T for F27 sample in concentrations ranging from 1.98 to 22 mg mL^−1^.

We used small‐angle X‐ray scattering (SAXS) and small‐angle neutron scattering (SANS) measurements to study self‐assembly of the particles in dispersion at room temperature. The experimental setup for SAXS and SANS measurements is depicted in (**Figure** **2a**). In this geometry, we detect the scattering along the magnitude of the scattering vector $Q\;=\;\frac{4\pi \;\sin\theta }{\lambda }$ where 2θ is the angle between the incident and scattered neutron beam, and λ is the incident X‐ray or neutron wavelength.

SAXS measurements experimentally probe the effect of size, concentration and applied field in self‐assembly of IONPs at room temperature (Figure S8, Supporting Information). SAXS measurements of F05 and F10 IONPs at 0.9 T show no self‐assembly even at the highest concentration, in agreement with N* < 1 (Figure S9, Supporting Information). The radially averaged SAXS data at 0 T (Figure 2c) for F20 and F24 samples are well described by a model of spherical form factor F(Q) (schematic in Figure 2b top) for noninteracting IONPs. The diameter, shape and size distribution obtained from SAXS data refinements agree well with TEM results. However, F27 IONPs at 0 T (Figure 2c) cannot be properly described by spherical form factor, due to the correlation peak in radially averaged data. This indicates that F27 IONPs aggregates even at 0 T. In order to quantify self‐assembly of F27 IONPs we fit the data to a linear pearl model (schematic in Figure 2b bottom). The model describes *N* spheres of radius *R* linearly joined by straight strings of negligible thickness.^[^ ^33^ ^]^ The fitting parameters are diameter *D* and edge separation parameter *ℓ* = *d*_ctc_ − *D*, where *d*_ctc_ is the center‐to‐center distance between the IONPs. *D* = 27(2) nm is obtained from this fit, which agrees well with TEM and SEM results, with *ℓ* = 9.7 nm for 0 T data. This result suggests that self‐assembly of F27 IONPs is best described by formation of chains even at zero field. When 0.9 T magnetic field is applied, the correlation peak becomes more intense compared to 0 T (Figure 2d). We further investigate the effect of varying concentration at 0.9 T for F27 IONPs (Figure 2e). Even when the dispersion is diluted by a factor of 10, the correlation peak is clearly visible, meaning that dilution did not disintegrate chains once they are formed. Since the applied field of 0.9 T used in SAXS experiments is above the saturation field, the magnetic moments of IONPs are aligned along the field. Analogous to the spinning forces that aligned the IONPs on a substrate (Figure 1d), the magnetic interactions between IONPs and applied field drive formation of chains. Surprisingly, SAXS experiments found no self‐assembly for F20 IONPs at 0.9 T, despite N* > 1 (Figure 2b).

We used SANS measurements to explore magnetic interactions between IONPs and to unambiguously determine the ligand shell thickness using selective isotope substitution (Figures S10 and S11, Supporting Information). A core–shell form factor was used to fit the data. The core size was fixed to the value obtained from SAXS and TEM, while shell thickness was refined. The shell thickness of 1.3(4) and 1.7(1) nm was obtained for F20 and F27 samples at 0 T, respectively. Both values are smaller than the nominal length of fully stretched oleic acid (OA) ligand, due to the bending conformity of the OA shell. Similar to SAXS patterns the 2D SANS patterns of F20 sample is isotropic even in the highest field of 2.2 T, indicating no self‐assembly, despite N* ≫ 1 (Figure S12, Supporting Information).

Thermogravimetric analysis (TGA) of F20 and F27 samples (Figure S13, Supporting Information) indicates that F20 sample has an 80% mass fraction of organic materials as compared to only 50% in F27 sample. The excess of organic materials in F20 sample, presumably in the form of micelles attached to shell or free radicals, can induce large repulsive forces competing with attractive dipolar ones. In an actual in vivo environment, the IONPs could be subject to various forces like electrostatic interactions, and additional repulsive forces which were not considered in Langevin simulations.

**Figure 3:**
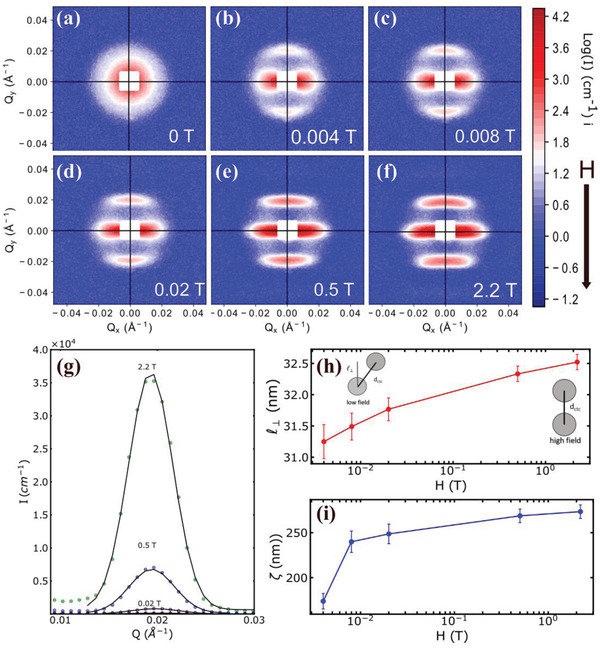
a–f) 2D SANS patterns of F27 from 0 to 2.2 T. g) 2D SANS patterns are divided into sectors of width 15° with sector centers at α  =  0°, where α is the angle between scattering vector *Q* and field direction *H*. The fits to Gaussian function are in solid black lines. h) Projection of the center‐to‐center distance (ℓ_⊥_) between the IONPs and i) correlation length ζ as a function of field. The solid lines are guide for eyes. The schematics show the bending of chains at low and high fields.

2D SANS pattern of F27 sample show intensity peaks around *Q*_*y*_ ≈ ±0.02 Å^−1^ even at low field of 0.004 T (**Figure** **3a**). Increasing the magnitude of the applied magnetic field changes the 2D SANS pattern of F27 IONPs from curved and diffuse horizontal stripes at low fields into sharp straight stripes at high fields (Figure 3a–f). Importantly, the field‐induced patterns revert back to their original state by removing the magnetic field. The radially averaged data shown in Figure 2 indicates the presence of chains, thus 2D anisotropic SANS data is divided into sectors parallel to the applied field *H* to reveal the structural details of these chains.

The correlation peak observed in the integrated intensity of the sector parallel to *H* is modelled with a Gaussian function in order to obtain the peak position and width (Figure 3g).

The peak position corresponds to the projection of the center‐to‐center distance between the IONPs on the field axis $\left(l_{⊥}=\;\frac{2\pi }{Q}\right)$. The width of the peak describes the correlation length given by ζ$=\;\frac{2\pi }{W}$, where *W* is the width of the correlation peak, related to the chain length. The dependence of both parameters on field magnitude is depicted in Figure 3h,i. With increasing field, the projected distances are found to lengthen as the chains straighten. The distance eventually becomes a constant at *H* ≥ 0.5 T, because the straight chains are in their most energetically favorable configuration.

The effective correlation length obtained at 2.2 T is ≈270 nm and contains about 8–9 individual IONPs. Despite the intense attraction, there is no continuous growth of chains as predicted by the Langevin simulations. A similar equilibrium state was previously observed for Co NPs with chain lengths of ≈65 nm containing 3–4 particles.^[^ ^28^ ^]^ Indeed, the chain lengths in solution of F27 IONPs are much shorter than those observed for the same particles deposited on a substrate (Figure 1c,d). The flexibility of chains in dispersion can be controlled by tuning the magnetic field. At higher applied fields, the strong interaction of the dipoles with magnetic field overcomes the dipole–dipole interactions between IONPs and disentangle the dipolar chains into straight chains.

**Figure 4:**
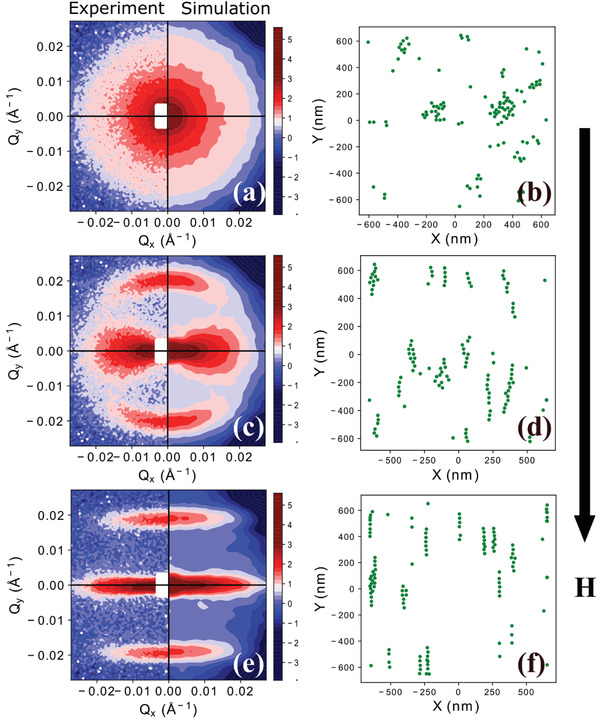
a–f) D experimental SANS and simulated patterns with corresponding real‐space positions of IONPs at 0 T (a,b), 0.006 T (c,d), and 2.2 T (e,f). The field direction is along the vertical direction, as shown by an arrow.

SANS data sector analysis and radially averaged SAXS/SANS data modelling using a linear pearl model both indicate the formation of tortuous chains induced by field that lengthen and straighten with increasing field. In order to gain insight into the real‐space distribution of IONPs in solution, we employed RMC simulations to analyze 2D SANS data. The core and shell parameters obtained from SAXS and SANS were used for defining the size of the particles in the box. The number of particles in the box was limited to 300. We match the experimental concentration by changing the size of the box. Thus, we compared RMC simulated and experimental 2D patterns by clipping the latter and putting both on the same scale. The 2D experimental and simulation patterns (**Figure** **4**) are shown for 50% d‐toluene contrasted data at a sample‐to‐detector distance of 14 m. At this distance, we have access to low *Q*, which contains high‐resolution data of the structure factor. At zero field, the small clusters of F27 IONPs are clearly visible (Figure 4). The direction of the effective dipolar field experienced by a single IONP is determined by random orientation and arrangements of its neighbors. On application of a field *H* ≤ 0.5 T, the direction of the IONPs dipolar field is in competition with the effective dipolar field from the clusters forcing more particles to align with the field and extend into ordered chains. As seen in Figure 4d, when *H* = 0.006 T there are linear chains bent along the field axis and some chains bent along the chain axis. When the field is above 0.5 T which is larger than the saturation field, straight chains are formed (Figure 4f). In agreement with the 1D SAXS/SANS analysis, we found an equilibrium state with finite chains and no exponential or power law increase in chain lengths with concentration.

## 3 Conclusion

The use of IONPs for specific in vivo applications depends on accurate determination of their control parameters for manipulation in applied magnetic field. Experimental measurements of size, composition, local crystal structure of IONPs and their interaction with the field, indicated the vital role of dipole–dipole interactions. In particular, we studied formation of flexible nanochains by 27 nm IONPs and their behavior in magnetic field. The length of the chains is ultimately controlled by a magnitude of applied magnetic field and it can be reversed. A powerful combination of scattering methods and RMC simulations enables studies on field‐driven self‐assembly of IONPs with more complex geometries than spherical particles. Real‐space visualization of self‐assembly of IONPs in solution provided by this combination is a key component for facilitating the development of novel biomedical applications. Future experiments using phosphate‐buffer saline in a microfluidics device driven by pulsatile flow could be a step towards approaching a more realistic biomedical environment.

## 4 Experimental Section

### Samples

Monodisperse IONPs with a diameter of 20 nm (F20) and 27 nm (F27) were synthesized using an extended LaMer mechanism described elsewhere.^[^ ^29^ ^]^ Commercially synthesized IONPs in solution with a diameter of 5 nm (F5) and 10 nm (F10) were obtained from NN labs and 24 nm (F24) from Ocean Nanotech. All IONPs were further coated with a shell of the oleic acid surfactant to prevent agglomeration. Reference powder of IONPs (F50) with a wide size distribution of 50–100 nm was obtained from Sigma Aldrich.

### Microscopy

TEM measurements were carried out in the Ernst Ruska‐Centre at Forschungszentrum Jülich with a Philips CM20 TEM and FEI Tecnai G2 F20 using an accelerating voltage of 200 kV.^[^ ^32^ ^]^

The SEM studies were performed on dried and spin‐coated IONPs using a Hitachi SU8000 instrument at 20 kV of accelerating voltage. About 25 µL of highly diluted solution of F27 IONPs was deposited on a n type (111) silicon substrate. The drop‐cast samples were allowed to dry out at ambient conditions. The spin‐coated samples were prepared by coating IONPs with speed of 30 rps in 1 min.

### Magnetization Measurements

DC magnetization measurements were carried out at temperatures from 5 to 350 K and in fields as large as 7 T using a Physical Property Measurement System (PPMS). The samples were dispersed in paraffin and sealed inside plastic capsules, which were inserted into PPMS using a brass rod. The standard field‐cooled (FC) and zero‐field‐cooled (ZFC) measurements at 0.01 T were used to define *T*_B_.

### X‐ray and Neutron Scattering

SAXS measurements were performed at GALAXI beamline in Forschungszentrum Jülich.^[^ ^33^ ^]^ The samples were dispersed in toluene and filled in borosilicate capillaries of 2 mm in diameter with a wall thickness of 0.05 mm. The wavelength was 1.34 Å and sample‐to‐detector distance of 3.5 m was used in order to cover *Q*‐range of 0.004–0.3 Å^−1^. The samples were studied in different concentrations ranging from 0.07 to 25 mg mL^−1^. All samples were measured in a magnetic field of either 0 or 0.9 T, perpendicular to the incoming beam. SAXS data was analyzed using the SASView software package.

The synchrotron X‐ray pair distribution function (PDF) measurements were carried out at the beamline MS‐X04SA at Swiss Light Source (SLS), Paul Scherrer Institute in Switzerland,^[^ ^34^ ^]^ and at P02.1 beamline at DESY in PETRA III facility in Hamburg, Germany.^[^ ^35^ ^]^ The following wavelengths were used: 0.432 Å (SLS) and 0.207 Å (DESY). The standard LaB_6_ and Ni bulk powders were measured to estimate the resolution of each instrument.

SANS experiments were carried out at the KWS‐1 instrument operated by the Jülich Centre for Neutron Science (JCNS) at Heinz Maier‐Leibnitz Zentrum (MLZ) in Garching, Germany.^[^ ^36^, ^37^ ^]^ The incident neutron wavelength, λ was fixed to 5 Å ($\frac{\Delta \lambda }{\lambda }=\;10\%)$. The IONPs in solutions were filled in 1 mm thick quartz Hellma cells of type 110 for room temperature. The sample‐to‐detector distances were 14, 8, and 2 m and thus provided a wide *Q*‐range coverage of 0.007–0.5 Å^−1^.

### Reverse Monte Carlo Simulations

RMC simulations were used to model 2D SANS data collected with unpolarized neutrons. Initially, an ensemble of *N* particles were distributed randomly within a box with dimensions, *L*  =  2π/*Q*_min_ where *Q*_min_ is the minimum *Q* value on the detector, outside the beamstop. The simulated intensity is calculated as

(1) $$\begin{array}{c} I_{\text{sim}}\;\left(Q\right)={\left|\overset{N}{\underset{j=1}{\sum }}\;F_{c-\text{shell}}\exp\left(-iQ.r_{j}\right)\;\right|}^{2} \end{array}$$

where, *r_j_* is the position of the *j*th particle.

Periodic boundary conditions were assumed for particle‐wall interactions and particle collision detection was accounted for. Each Monte Carlo step was performed by choosing a random particle and moving it randomly by one of three processes: linear move, jump, and orbital motion. The movement is accepted only if it resulted in decrease of discrepancy between the experimental and simulated 2D SANS data. The number of IONPs in simulations was set for *N* =  300 and the total number of Monte Carlo cycles was 100 steps per particle. The 2D RMC results were smeared with the instrument resolution function for a given sample‐to‐detector distance.

## Conflict of Interest

The authors declare no conflict of interest.

## Supporting information

Supporting Information

## Data Availability

Data available on request from the authors.

## References

[1] Z. Zhou , L. Yang , J. Gao , X. Chen , Adv. Mater. 2019, 31, 1804567. 10.1002/adma.201804567 PMC6392011 30600553

[2] Q. Pankhurst , S. Jones , J. Dobson , J. Phys. D: Appl. Phys. 2016, 49, 501002.

[3] H. Nosrati , M. Salehiabar , M. Fridoni , M.‐A. Abdollahifar , H. Kheiri Manjili , S. Davaran , H. Danafar , Sci. Rep. 2019, 9, 7173. 31073222 10.1038/s41598-019-43650-4 PMC6509211

[4] Z. Tay , P. Chandrasekharan , A. Chiu‐Lam , D. Hensley , R. Dhavalikar , X. Zhou , E. Yu , P. Goodwill , B. Zheng , C. Rinaldi , S. Conolly , ACS Nano 2018, 12, 3699. 29570277 10.1021/acsnano.8b00893 PMC6007035

[5] C. Shasha , K. Krishnan , Adv. Mater. 2021, 33, 1904131. 10.1002/adma.201904131 PMC7746587 32557879

[6] A. Amirfazli , Nat. Nanotechnol. 2007, 2, 467. 18654342 10.1038/nnano.2007.234

[7] Z. Li , F. Yang , Y. Yin , Adv. Funct. Mater. 2019, 30, 1903467.

[8] W. Wu , Z. Wu , T. Yu , C. Jiang , W. Kim , Sci. Technol. Adv. Mater. 2015, 16, 023501. 27877761 10.1088/1468-6996/16/2/023501 PMC5036481

[9] J. Perez , F. Simeone , Y. Saeki , L. Josephson , R. Weissleder , J. Am. Chem. Soc. 2003, 125, 10192. 12926940 10.1021/ja036409g

[10] M. Klokkenburg , B. Erné , A. Wiedenmann , A. Petukhov , A. Philipse , Phys. Rev. E 2007, 75, 051408. 10.1103/PhysRevE.75.051408 17677066

[11] M. Klokkenburg , B. Erné , J. Meeldijk , A. Wiedenmann , A. Petukhov , R. Dullens , A. Philipse , Phys. Rev. Lett. 2006, 97, 185702. 17155554 10.1103/PhysRevLett.97.185702

[12] Z. Fu , Y. Xiao , A. Feoktystov , V. Pipich , M. Appavou , Y. Su , E. Feng , W. Jin , T. Brückel , Nanoscale 2016, 8, 18541. 27782247 10.1039/c6nr06275j

[13] L. Wang , A. Qdemat , O. Petracic , E. Kentzinger , U. Rücker , F. Zheng , P. Lu , X. Wei , R. Dunin‐Borkowski , T. Brückel , Phys. Chem. Chem. Phys. 2019, 21, 6171. 30821806 10.1039/c9cp00302a

[14] U. Cheang , M. Kim , J. Nanopart. Res. 2015, 17, 145.

[15] S. Corr , S. Byrne , R. Tekoriute , C. Meledandri , D. Brougham , M. Lynch , C. Kerskens , L. O'Dwyer , Y. Gun'ko , J. Am. Chem. Soc. 2008, 130, 4214. 18331033 10.1021/ja710172z

[16] B. Su , Y. Wu , Y. Tang , Y. Chen , W. Cheng , L. Jiang , Adv. Mater. 2013, 25, 3968. 23716138 10.1002/adma.201301003

[17] N. Ilawe , M. Oviedo , B. Wong , J. Mater. Chem. C 2018, 6, 5857.

[18] L. Chen , B. Su , L. Jiang , Chem. Soc. Rev. 2019, 48, 8. 30444250 10.1039/c8cs00703a

[19] Z. Tang , N. Kotov , Adv. Mater. 2005, 17, 951.

[20] H. Wang , Y. Yu , Y. Sun , Q. Chen , Nano 2011, 06.

[21] Y. Min , M. Akbulut , K. Kristiansen , Y. Golan , J. Israelachvili , Nat. Mater. 2008, 7, 527. 18574482 10.1038/nmat2206

[22] P. Gennes , P. Pincus , Phys. Kondens. Mater. 1970, 11, 189.

[23] J. Tavares , J. Weis , M. Telo da Gama , Phys. Rev. E 1999, 59, 4388.

[24] K. Butter , P. Bomans , P. Frederik , G. Vroege , A. Philipse , J. Phys.: Condens. Matter 2003, 15, S1451. 10.1038/nmat811 12612691

[25] M. Klokkenburg , C. Vonk , E. Claesson , J. Meeldijk , B. Erné , A. Philipse , J. Am. Chem. Soc. 2004, 126, 16706. 15612692 10.1021/ja0456252

[26] S. Singamaneni , V. Bliznyuk , C. Binek , E. Tsymbal , J. Mater. Chem. 2011, 21, 16819.

[27] J. Mikšátko , D. Aurélio , P. Kovaříček , M. Michlová , M. Veverka , M. Fridrichová , I. Matulková , M. Žáček , M. Kalbáč , J. Vejpravová , Nanoscale 2019, 11, 16773. 31309957 10.1039/c9nr03531a

[28] M. Barrett , A. Deschner , J. Embs , M. Rheinstädter , Soft Matter 2011, 7, 6678.

[29] E. Vreeland , J. Watt , G. Schober , B. Hance , M. Austin , A. Price , B. Fellows , T. Monson , N. Hudak , L. Maldonado‐Camargo , A. Bohorquez , C. Rinaldi , D. Huber , Chem. Mater. 2015, 27, 6059.

[30] G. Goya , T. Berquó , F. Fonseca , M. Morales , J. Appl. Phys. 2003, 94, 3520.

[31] J. Faraudo , J. Andreu , J. Camacho , Soft Matter 2013, 9, 6654.

[32] M. Luysberg , M. Heggen , K. Tillmann , J. Large‐Scale Res. Facil. 2016, 2, 77.

[33] E. Kentzinger , M. Krutyeva , U. Rücker , J. Large‐Scale Res. Facil. 2016, 2, 61.

[34] P. Willmott , D. Meister , S. Leake , M. Lange , A. Bergamaschi , M. Böge , M. Calvi , C. Cancellieri , N. Casati , A. Cervellino , Q. Chen , C. David , U. Flechsig , F. Gozzo , B. Henrich , S. Jäggi‐Spielmann , B. Jakob , I. Kalichava , P. Karvinen , J. Krempasky , A. Lüdeke , R. Lüscher , S. Maag , C. Quitmann , M. Reinle‐Schmitt , T. Schmidt , B. Schmitt , A. Streun , I. Vartiainen , M. Vitins , X. Wang , R. Wullschleger , J. Synchrotron Radiat. 2013, 20, 667. 23955029 10.1107/S0909049513018475 PMC3747948

[35] A. Dippel , H. Liermann , J. Delitz , P. Walter , H. Schulte‐Schrepping , O. Seeck , H. Franz , J. Synchrotron Radiat. 2015, 22, 675. 25931084 10.1107/S1600577515002222 PMC4416682

[36] A. Feoktystov , H. Frielinghaus , Z. Di , S. Jaksch , V. Pipich , M. Appavou , E. Babcock , R. Hanslik , R. Engels , G. Kemmerling , H. Kleines , A. Ioffe , D. Richter , T. Brückel , J. Appl. Crystallogr. 2015, 48, 61.

[37] H. Frielinghaus , A. Feoktystov , I. Berts , G. Mangiapia , J. Large‐Scale Res. Facil. 2015, 1, 28.

