# Supplementary material for: Unravelling Magnetic Nanochain Formation in Dispersion for In Vivo Applications
Source: Adv Mater. 2021 May 7;33(24):2008683. doi: 10.1002/adma.202008683 (PMC11468943; doi:10.1002/adma.202008683)
Supplement: Supplementary file 1 — Supporting Information [file ADMA-33-2008683-s001.pdf]

© 2021 Wiley-VCH GmbH

ADVANCED MATERIALS

Supporting Information

for *Adv. Mater.,* DOI: 10.1002/adma.202008683

Unravelling Magnetic Nanochain Formation in Dispersion for In Vivo Applications

*Nileena Nandakumaran, Lester Barnsley, Artem Feoktystov, Sergei A. Ivanov, Dale L. Huber, Lisa S. Fruhner, Vanessa Leffler, Sascha Ehlert, Emmanuel Kentzinger, Asma Qdemat, Tanvi Bhatnagar-Schöffmann, Ulrich Rücker, Michael T. Wharmby, Antonio Cervellino, Rafal E. Dunin-Borkowski, Thomas Brückel, and Mikhail Feygenson**

WILEY-VCH

© 2021 Wiley-VCH GmbH

Supporting Information

Unravelling Magnetic Nanochain Formation in Dispersion for In-Vivo Applications

*Nileena Nandakumaran, Lester Barnsley, Artem Feoktystov, Sergei A. Ivanov, Dale L. Huber, Lisa S. Fruhner, Vanessa Leffler, Sascha Ehlert, Emmanuel Kentzinger, Asma Qdemat, Tanvi Bhatnagar-Schöffmann, Ulrich Rücker, Michael T. Wharmby, Antonio Cervellino, Rafal E. Dunin-Borkowski, Thomas Brückel, Mikhail Feygenson**

A.Transmission Electron Microscopy (TEM) for F05, F10, F24 samples

Figure S1 shows representative TEM images of the commercial obtained particles F05, F10 and F24 samples with a derived polydispersity.

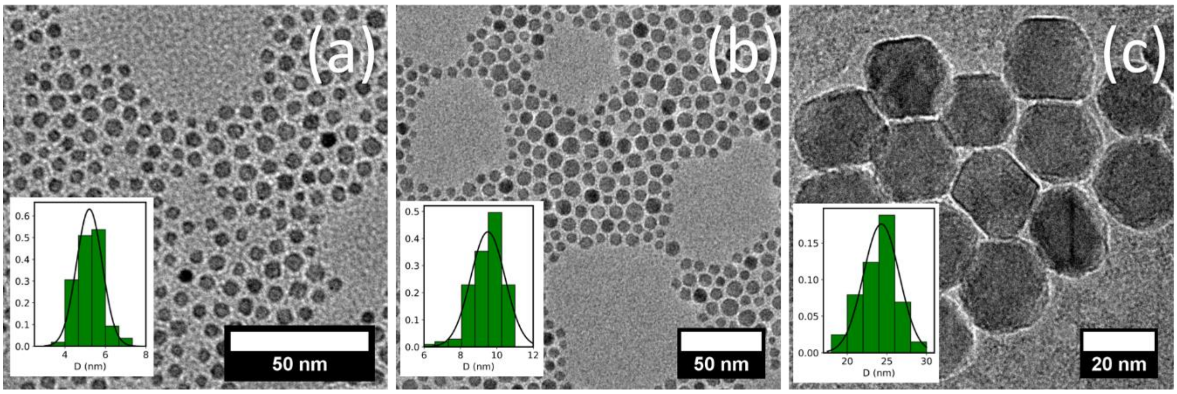

Figure S1: TEM images of (a) F05 (b) F10 (c) F24 samples with insets showing their size distribution

B. X-ray PDF measurements

We carried out synchrotron x-ray PDF measurements in order to probe their composition and local crystal structure of IONPs. It is well documented, that several phases of iron oxide can coexist in IONPs *[1, 2, 3, 4, 5, 6]*. The most probable phases are antiferromagnetic FeO (wüstite), ferrimagnetic Fe_3O_4 (magnetite), antiferromagnetic α-Fe_2O_3 (hematite) and ferrimagnetic γ-Fe_2O_3 (maghemite). The bulk powders of all these materials were measured as a reference under the same experimental conditions as IONPs.

The quality of the fits is shown in **Figure. S2**. The best fit of the experimental data for F20 and F27 samples was obtained by the model including two phases: maghemite (ISCD-79196) and magnetite (ISCD-65339). The scale factor, lattice constant, atomic displacement parameters and linear atomic correlation factor δ_l were refined for each phase. The best model for F20 includes a mixed composition of 23 % maghemite and 77 % magnetite (mass fractions), while the model for F27 is a mixture of 48 % maghemite and 52 % magnetite. The lattice constants of two phases in the IONPs are reduced compared to their bulk counterparts. Such finite-size effects have been previously reported for metallic IONPs *[7]*. During refinements of PDF data for F20 and F27 particles, the coordinates of all atoms were fixed to the bulk values. This result further reinforces our conclusion from TEM studies that F20 and F27 IONPs are of the high crystalline quality, however deviations of the model from the experimental PDF data at r < 12 Å might indicate existence of the local disorder or anti-phase boundary conditions.

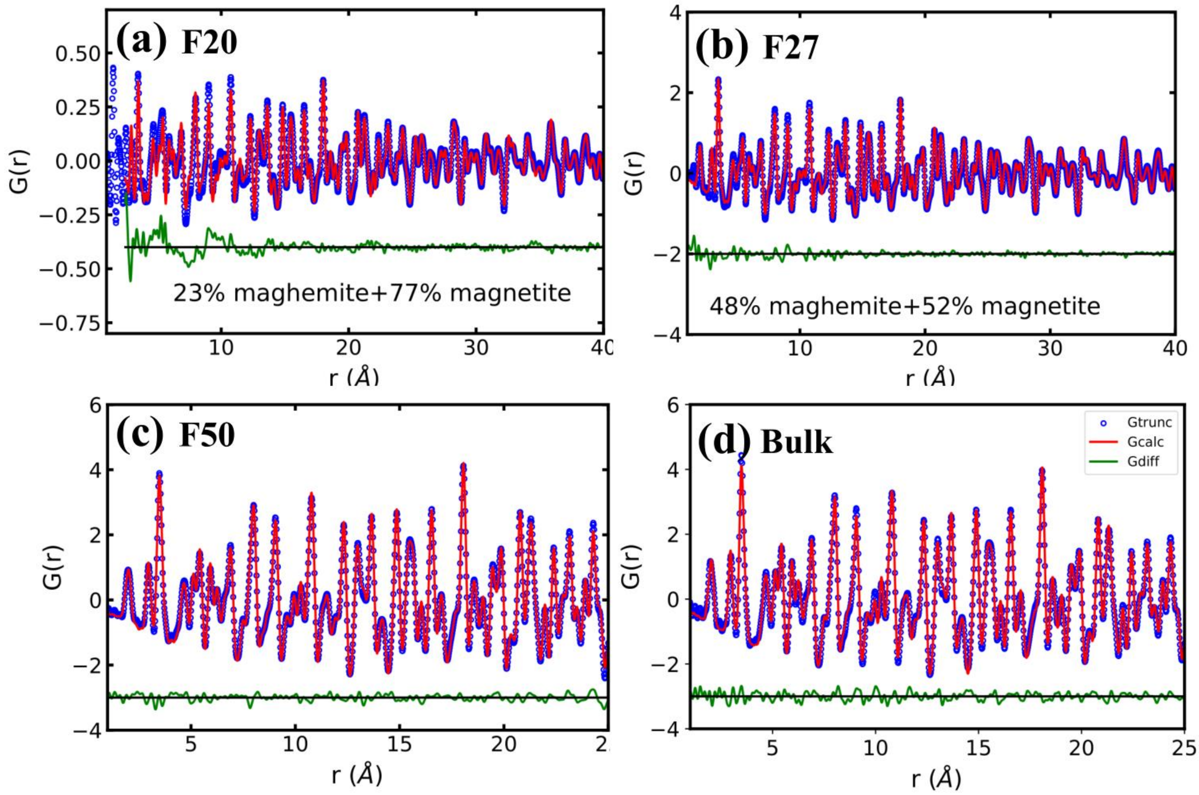

Figure S2: The best refinements of PDF data for (a) F20 (b) F27 (c) F50 IONPs and (d) magnetite powdered bulk, with experimental data in open circles, calculated pattern in red and difference curve in green.

B. DC magnetization measurements

The overview of temperature-dependent DC magnetization measurements for F05, F10, F24 and F50 IONPs is shown in **Figure S3**.
The T_B of IONPs is related to the energy barrier ΔE, separating two easy directions of magnetization via Arrhenius's law *[8]*.

$$T_B = \frac{\Delta E}{25k_B} \tag{1}$$

where, k_B is the Boltzmann constant. For non-interacting IONPs, the energy barrier is $\Delta E = KV$, where K is the anisotropy constant and V is the magnetic volume of the MNP. We calculated T_B for a given size using **Eq. 1** and assuming bulk anisotropy constants for magnetite (K = 1.3 x 10^4 J/m^3) and maghemite (K = 10^4 J/m^3). **Figure S4** compares M(T) of F27 IONPs at three different concentrations to reveal no change in blocking temperature even at extreme dilutions. **Figure S5** compares M(T) of the F05 and F10 IONPs at two different concentrations. **Figure S6** shows the comparison of calculated and experimentally measured T_B. Clearly, the experimental $T_B$is higher than calculated one for all IONPs. There are several reasons for such discrepancy. The anisotropy constant in IONPs can be higher compared to the bulk values, as it was observed for Co IONPs *[9]*.

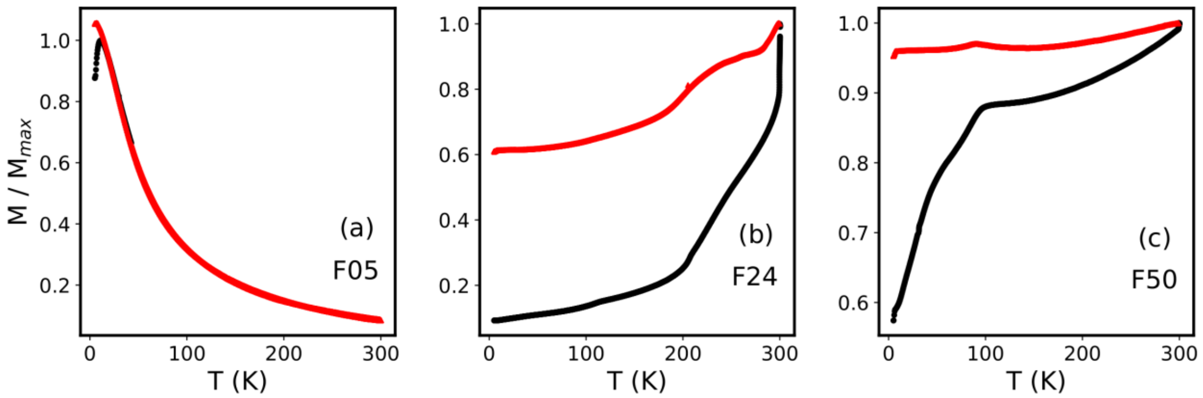

Figure S3: ZFC (●) and FC (▲) magnetizations as a function of temperature for (a) F05 (b) F24 and (c) F50 IONPs.

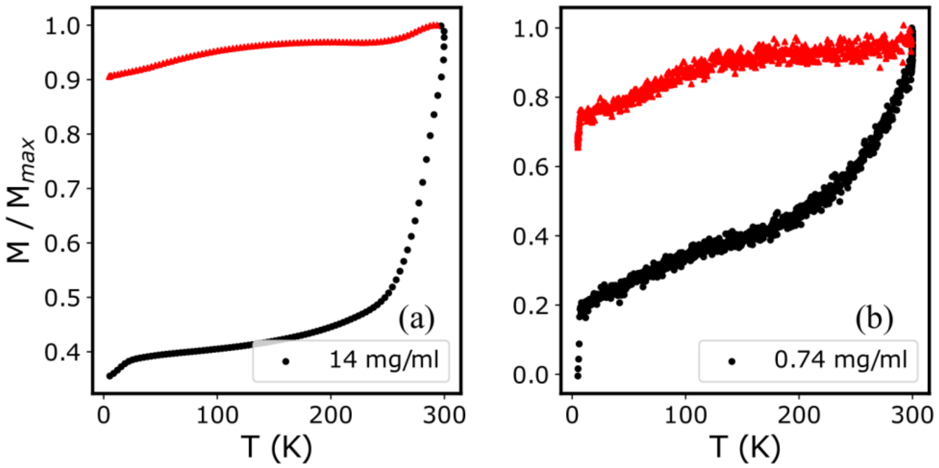

Figure S4: ZFC (●) and (▲) FC magnetizations for F27 at (a) 14 mg/ml and (c) 0.74 mg/ml

However, we found no previous reports of increased K for magnetite or maghemite IONPs. We can also reject the explanation of increased T_B due to higher magnetic volume of our IONPs, because SANSPOL measurements confirmed very little variation between nuclear and magnetic volumes of particles. The most likely explanation of increased T_B in our samples is the presence of magnetic dipole-dipole interactions, even when they are diluted in paraffin. The energy of magnetic dipole–dipole interactions between IONPs E_{int} , increases the energy barrier ΔE = KV + E_{int} and consequently T_B *[10, 11]*.

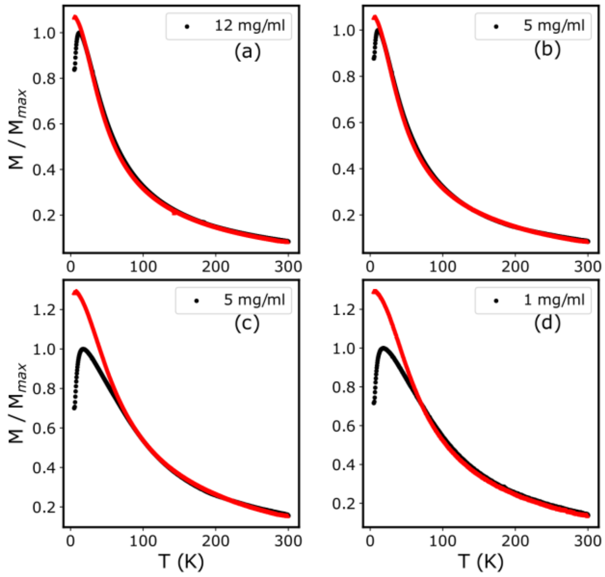

Figure S5: ZFC (●) and FC (▲) magnetizations for F05 at (a) 12 mg/ml, (b) 5 mg/ml. The same for F10 at (c) 5 mg/ml and (d) 1 mg/ml

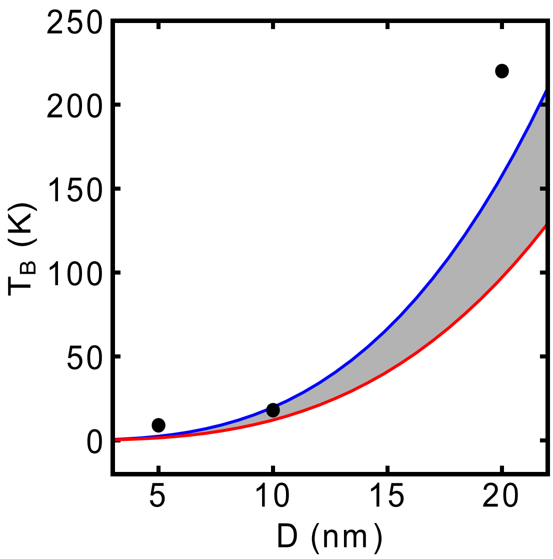

Figure S6: The experimental TB (●) as a function of particle diameter. The blue and red solid lines are calculated TB using K for bulk magnetite and maghemite, respectively

C. Calculation of N*

$\gamma = \frac{\mu_0 \mu^2}{2\pi k_B T D^3}$, where $\mu = \frac{M\pi D^3}{6}$

$\phi_o = 22\ mg/mL$

N* = $\sqrt{\phi_0 e^{\gamma - 1}}$

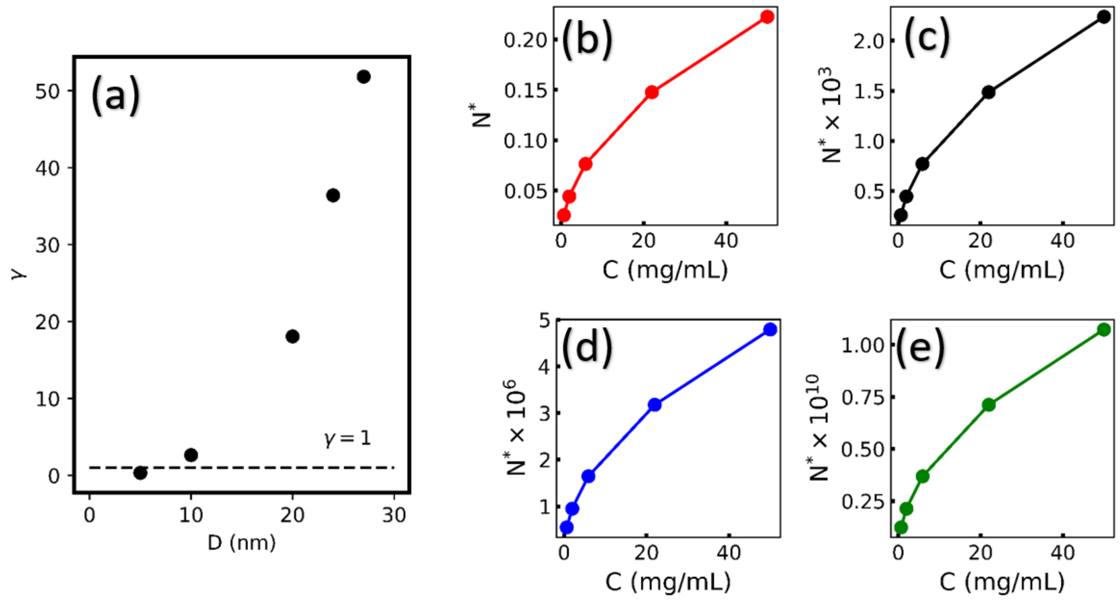

Figure S7: (a) γ calculated for different sizes of IONPs. N* calculated for (b) F10 (c) F20 (d) F24 (e) F27 as a function of concentration

D. SAXS measurements

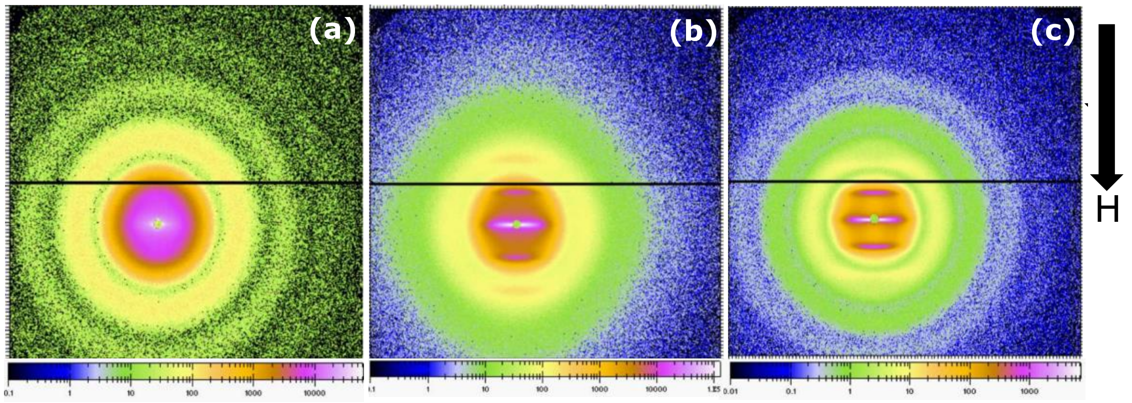

Figure S8: The 2D detector image showing SAXS data collected in 0.9 T magnetic field of (a) F20, (b) F24 and (c) F27 samples with similar concentrations of 20 mg/ml. The color scale is intensity in log scale. The black arrow shows the direction of applied field.

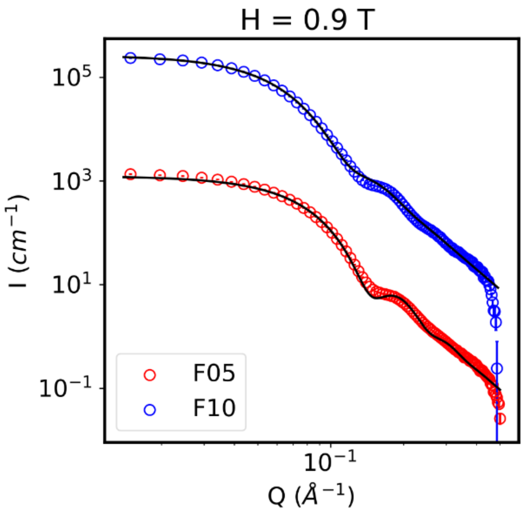

Figure S9: SAXS data of F05 and F10 at H=0.9 T, where solid black lines are fit to the spherical form factor

F05 and F10 IONPs at H= 0.9 T show no anisotropy. The data is fit to spherical form factor (**Figure S9**).

E. Ligand Thickness for F20 and F27

SANS data of the F20 particles do not show any structural peaks, so we used a core-shell form factor to fit the data. The core radius was fixed to the value obtained from SAXS data refinement and the shell thickness was refined. The refined shell thickness was 1.0(1) nm for the SANS data with 100% h-toluene contrast (**Figure S10a**). We repeated the refinements for 100 % d-toluene contrast (core contrast). The refined shell thickness was 1.3(4) nm (**Figure S10b**). We choose to use the thickness obtained from d-toluene due to its sharper contrast with core.

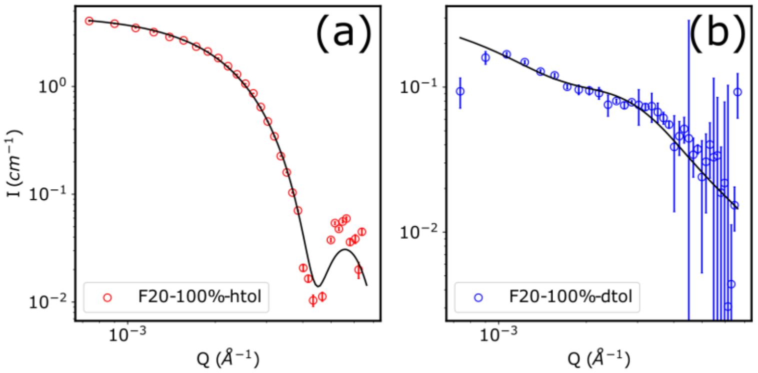

Figure S10: F20 MNPs dispersed in (a) h-toluene (b) d-toluene. Solid line corresponds to the fit using the form factor for the spherical core-shell nanoparticles

SANS data of F27 particles was also fit to the core-shell model at higher Q with four different contrasts (**Figure S11**). The thickness of shell is determined by fixing the radius of the core, polydispersity and refining the thickness of the shell. The determined thickness t = 1.7(1) nm for contrasts calculated at 0 T.

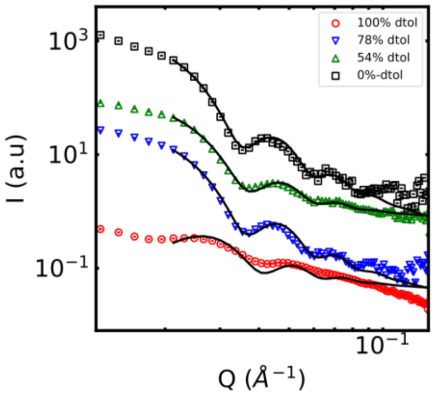

Figure S11: SANS measurements of F27 sample prepared in different contrasts (100 %, 78%, 54 % and 0% d-toluene) and the solid lines are fit to core-shell model

F. SANS measurements of F20 IONPs

Figure S12a shows 2D isotropic SANS pattern for F20 sample at 2.2 T. Radially averaged SANS data for F20 and F27 samples emphasize the formation of chains in the latter (**Figure S12b**).

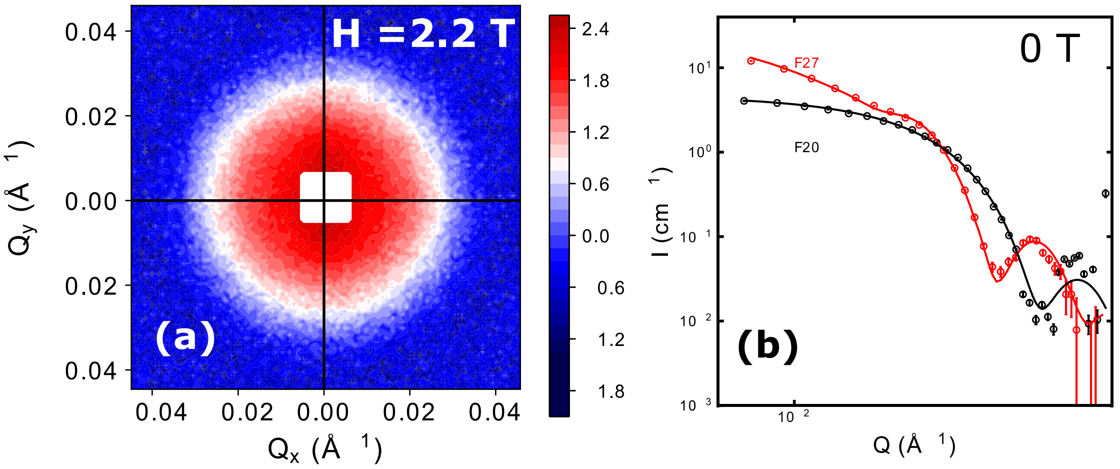

Figure S12: (a) 2D SANS scattering pattern in F20 at 2.2 T. (b) Radially averaged intensity of F20 and F27 at 0 T. The solid lines are fit to spherical model for F20 and linear pearl model for F27.

G. TGA measurements

Thermal Gravimetric Analysis (TGA) measurements were carried at Forschungszentrum Jülich with NETZSCH TG 209F1 Libra. The dried powders of F20 and F27 IONPs were measured by heating the samples in nitrogen atmosphere up to 700 °C in alumina crucible at the rate of 5 °C/min. We do not observe a significant weight loss at the boiling point of OA (95 - 115 °C), indicating a negligible amount of free OA in both F20 and F27 samples. The significant weight loss occurred at around (380 - 430°C) due to complete combustion of organic material. The loss percent is significantly larger in F20 (~80 %) compared to F27 (~ 50 %) indicating a larger percentage of OA. The other losses at higher temperatures are due to a phase transition of the IONPs.

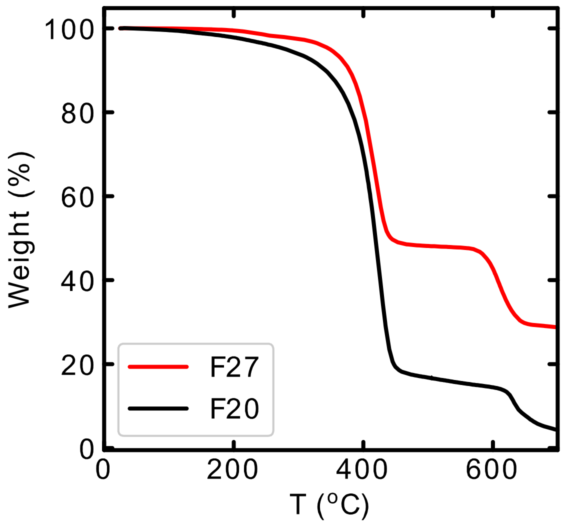

Figure S13: TGA of F20 and F27 samples

References

[1] Sun, X., Frey Huls, N., Sigdel, A., & Sun, S. (2012). Nano letters, 12(1), 246-251

[2] Sharma, S. K., Vargas, J. M., Pirota, K. R., Kumar, S., Lee, C. G., & Knobel, M. (2011). Journal of alloys and compounds, 509(22), 6414-6417.

[3] Kavich, D. W., Dickerson, J. H., Mahajan, S. V., Hasan, S. A., & Park, J. H. (2008). Physical Review B, 78(17), 174414.

[4] Swiatkowska-Warkocka, Z., Kawaguchi, K., Wang, H., Katou, Y., & Koshizaki, N. (2011). Nanoscale research letters, 6(1), 226

[5] Redl, F. X., Black, C. T., Papaefthymiou, G. C., Sandstrom, R. L., Yin, M., Zeng, H., & O'Brien, S. P. (2004). Journal of the American Chemical Society, 126(44), 14583-14599.

[6] Nogués, J., & Schuller, I. K. (1999). Journal of Magnetism and Magnetic Materials, 192(2), 203-232

[7] Page, K., Proffen, T., Niederberger, M., & Seshadri, R. (2010). Probing local dipoles and ligand structure in BaTiO3 nanoparticles. Chemistry of Materials, 22(15), 4386-4391.

[8] Chantrell, R. W., & Wohlfarth, E. P. (1983). Journal of magnetism and magnetic materials, 40(1-2), 1-11

[9] Chen, J. P., Sorensen, C. M., Klabunde, K. J., & Hadjipanayis, G. C. (1995). Physical Review B, 51(17), 1152

[10] Hansen, M. F., & Mørup, S. (1998). Journal of Magnetism and Magnetic Materials, 184(3), L262-274.

[11] Petit, C., Taleb, A., & Pileni, M. P. (1998). Advanced Materials, 10(3), 259-261
